# Motivational aspects among MEDical Students to Participate as first ResPONDer in out-of-hospital cardiac arrest (MEDSPOND): a pre-post educational intervention study

**DOI:** 10.1186/s12909-026-09445-8

**Published:** 2026-05-14

**Authors:** Julian Ganter, Lukas Fiebelkorn, Joachim Bansbach, Stefan Bushuven, Hans-Joerg Busch, Hartmut Buerkle, Sebastian Heinrich

**Affiliations:** 1 Department of Anesthesiology and Critical Care, Faculty of Medicine, Medical Center - University of Freiburg, University of Freiburg, Hugstetter Str. 55, Freiburg, 79106 Germany; 2 Training Center for Emergency Medicine (NOTIS e.V), Singen, Germany; 3 Institute of Medical Education, LMU University Hospital, LMU Munich, Munich, Germany; 4 Department of Emergency Medicine, Faculty of Medicine, University Hospital of Freiburg, University of Freiburg, Freiburg, Germany

**Keywords:** Emergency Medicine, Cardiac Arrest, Defibrillation, Smartphone Alerting System, Medical Teaching

## Abstract

**Background:**

Recent cardiopulmonary resuscitation (CPR) guidelines highlight the role of local, trained individuals in responding to out-of-hospital cardiac arrest (OHCA). In addition to healthcare professionals, medical students can be integrated into smartphone alerting systems (SAS) as potential locally active community first responders. However, there is a limited understanding of confounding factors which may stimulate or hinder student engagement. This study explores multiple factors to further support targeted recruitment and training strategies.

**Methods:**

This prospective pre-post study was integrated into an emergency medicine course with the clinical medical curriculum. Participants completed anonymized digital questionnaires before and after the course. The curriculum included basic life support and introduced the SAS *Region of Lifesavers*. The questionnaire focused on self-assessed preparedness, willingness to participate, and motivational factors related to community first responder engagement. Ethical approval was obtained (DRKS00034599).

**Results:**

Of 412 students invited to participate in pre- and post-course surveys, 93.2% (384) were matched for analysis. The cohort was 64% female, mean age of 23 years. Prior medical CPR training was present in 25% (95/384); only 13% (12/95) of these were active as first responders. Confidence in BLS increased, with self-rated skills rising from 4.9 to 7.6 (Likert 0–10); confidence regarding cardiac arrest response improved from 3.9 to 7.9 (both *p* < 0.001). Intention to join as first responders was noted in 80.2% of participants, mainly motivated by “willingness to help” (48.3%) and “the topic is important” (22.6%). The main barriers were lack of self-confidence (41.3%) and perceived personal stress (37.3%).

**Conclusion:**

This study highlights strong motivation among medical students and identifies practical training targeting on CPR confidence as a key determinant of enhanced first responder engagement. Pre-existing curricular emergency medicine courses offer a structured opportunity to promote awareness of first responder systems and to prepare students for potential future involvement.

**Trial registration:**

German Clinical Trials Register, DRKS00034599, date of registration: 16.12.2024.

**Supplementary Information:**

The online version contains supplementary material available at 10.1186/s12909-026-09445-8.

## Background

Out-of-hospital cardiac arrest (OHCA) represents a medical emergency in which time to intervention is a critical determinant of patient survival. In response to this urgency, current international resuscitation guidelines recommend the activation of nearby individuals trained in cardiopulmonary resuscitation (CPR) to initiate basic life support as early as possible [1, 2]. This recommendation is being implemented more and more widely, primarily through the use of smartphone alerting systems (SAS) [3]. These systems alert registered volunteers, who are classified as community first responders, who are situated in close proximity to the emergency [4]. The effectiveness of SAS is influenced by technological performance as well as by the density and availability of registered volunteers in the addressed area [5]. The present study refers to the SAS “Region of Lifesavers,” which is implemented in the region surrounding the University of Freiburg [6]. This smartphone-based first responder system is, among several SAS implemented nationwide [7], the largest system in Germany and is integrated into the respective emergency medical dispatch centers. In the event of an emergency call with suspected OHCA, nearby registered first responders are alerted via an algorithm-based smartphone application in parallel with standard emergency medical services (EMS) dispatch, using GPS-based localization, task-specific assignment, and integration of automated external defibrillator (AED) databases [8]. While paramedics, physicians and other healthcare professionals are commonly regarded as key participants in these systems, medical students who have completed structured curricula in emergency medicine training are likewise qualified to contribute meaningfully as first responders. Registration is voluntary and often restricted to individuals with verified medical qualifications and certified Basic Life Support (BLS) training [9]. Within the “Region of Lifesavers” system, medical students have been eligible for registration after completion of the first state examination (following the fourth semester) since the system’s initial implementation [10]. This eligibility has since been formally anchored in state legislation at the study location, allowing medical students to register in smartphone alerting systems after passing the first part of the medical licensing examination [11]. Given their medical background and proximity to clinical environments, medical students therefore may represent a valuable but still underutilized resource in strengthening first responder networks.

To effectively engage this identified group, it is crucial to understand the motivational factors that influence their willingness not only to perform CPR when present by chance, but also to actively participate as registered community first responders [12, 13]. However, existing research into these motivational factors among medical students remains scarce [14–16]. In order to align future educational interventions and emergency medicine curricula with the aim of encouraging volunteer participation in first responder programs [17], a systematic analysis of relevant motivational drivers and obstacles is required.

This study aims to investigate which motivational factors encourage medical students to register and participate as community first responders, and which perceived barriers may prevent such engagement. The overarching goal is to increase the number of registered first responders by identifying and understanding these factors in order to develop more targeted recruitment strategies in the future.

## Methods

### Study design and setting

The study design followed a prospective pre-post format and was fully embedded within the structure of the emergency medicine block course by using an anonymous questionnaire survey tool. At the very beginning of the first seminar session, all participants were invited to complete a survey by scanning a QR code displayed during the welcome presentation. This initial questionnaire, referred to as the pre-course survey, was completed during an extra time slot of five minutes to ensure that all students had the opportunity to respond simultaneously under comparable conditions. After completing the pre-course survey, the emergency medicine block course continued as per the standard curriculum. On the second day after the last seminar, the participants were given a second QR code. As before, students were invited to complete a digital questionnaire as part of the post-course survey. As the curricular two-day block course is offered repeatedly until all students have participated, the study procedure was applied uniformly across cohorts, with the pre-course survey administered during the welcome presentation and the post-course survey conducted at the conclusion of the course, between 10 February 2025 and 14 March 2025. This structure enabled a direct comparison of student responses before and after course participation.

### Emergency medicine course

The study was conducted within the framework of the emergency medicine course, a compulsory block course undertaken by medical students during their clinical training phase in the third year of medical training. All students had completed the first part of the medical licensing examination and received formal first aid training by the time they participated. Students attending this course were usually enrolled in their fifth or sixth semester (third year of medical training), depending on the cohort to which they were assigned. The block course is offered every year during February to March and is designed to accommodate students from both semesters in a rotating format. The course spans two consecutive days and is repeated over a period of approximately four weeks until the entire cohort has completed it. The structure alternates between theoretical seminars and practical skills sessions. Seminar groups consist of around 20 students and are further subdivided into smaller working groups during hands-on training.

The curriculum of the course covers core emergency medicine competencies, including BLS, trauma management, and other acute care topics, in accordance with national educational recommendations (NKLM 2.0 VII.4). Students are also introduced to the concept of SAS. In this context, the SAS *Region of Lifesavers*, which has been operational in the Freiburg region since 2018, is presented [6]. Informational flyers were distributed to students, outlining the registration process and essential requirements for participation. Upon successful completion of the emergency medicine course, students receive a certificate that can be used to verify their qualifications for registration as a community first responder within the *Region of Lifesaver* app.

### Questionnaire and data processing

The questionnaire was developed based on practice-relevant topics and enriched by existing literature on first responder engagement and motivation. Content validity was established through an iterative expert review process involving six independent professionals (all of whom were experts in emergency medicine, three of whom additionally had expertise in medical education), supplemented by an additional independent reviewer assessing overall questionnaire comprehensibility. Items were reviewed for relevance, clarity, and alignment with the study objectives, refined based on qualitative feedback, and finalized following a second consensus review. Technical implementation and data collection were carried out using the SoSci Survey platform (SoSci Survey GmbH, Germany), a web-based tool that complies with current data protection regulations. Its use was recommended by institutional data protection experts and its suitability for academic research. Data collection was conducted exclusively through this platform. All collected data were stored securely in accordance with data protection standards on institutional servers within the University Medical Center Freiburg. Participation in the survey required informed consent, which was obtained digitally from all participants prior to questionnaire access. The study adhered to all applicable data protection and ethical guidelines throughout its execution.

The questionnaire focused on self-assessed preparedness, willingness to participate, and motivational factors related to community first responder engagement. Both the pre- and post-course questionnaires are available in the supplementary material, provided in their original German version and in English translation.

### Statistical analysis and ethical approval

Descriptive data were analyzed using Microsoft Excel (Version Professional Plus 2024). Further analyses were conducted using IBM SPSS Statistics 29 (Armonk, NY, USA). For analyses regarding the question “Do you intend to participate as a first responder? (Single-Choice)”, responses were dichotomized by combining “No” as negative and both “Yes” and “I am already registered” as positive.

The descriptive analyses were conducted using absolute frequencies and percentages for categorical variables, and means with their respective standard deviations (SD) for continuous variables. Pre–post comparisons of participants’ assessments before and after the course were performed using paired-samples *t*-tests. Binary logistic regression analyses were carried out to examine associations between predictors and the binary outcome (all used items attached to supplement). Forward and backward stepwise selection procedures were applied using the likelihood ratio criterion. Both approaches resulted in identical final models. Multicollinearity was assessed using correlation matrices and variance inflation factors, with no indication of problematic collinearity. Missing data were handled via complete case analysis without imputation. Given the exploratory nature of the study, no formal adjustment for multiple testing was performed. Model fit was evaluated using the omnibus test and the Hosmer–Lemeshow test. Odds ratios (OR) with 95% confidence intervals (CI) are reported. All tests were considered significant for *p*-values ≤ 0.05. As an explorative study, we did not control for multiplicity. We excluded missing data listwise.

The trial was approved by the Ethics Committee of the Albert-Ludwigs-University Freiburg (No: 24-1441-S1) and registered with the German Register of Clinical Studies (No: DRKS00034599, 16.12.2024). Informed consent to participate was obtained from all of the participants. This study was conducted in accordance with the ethical principles of the Declaration of Helsinki.

## Results

**Fig. 1:**
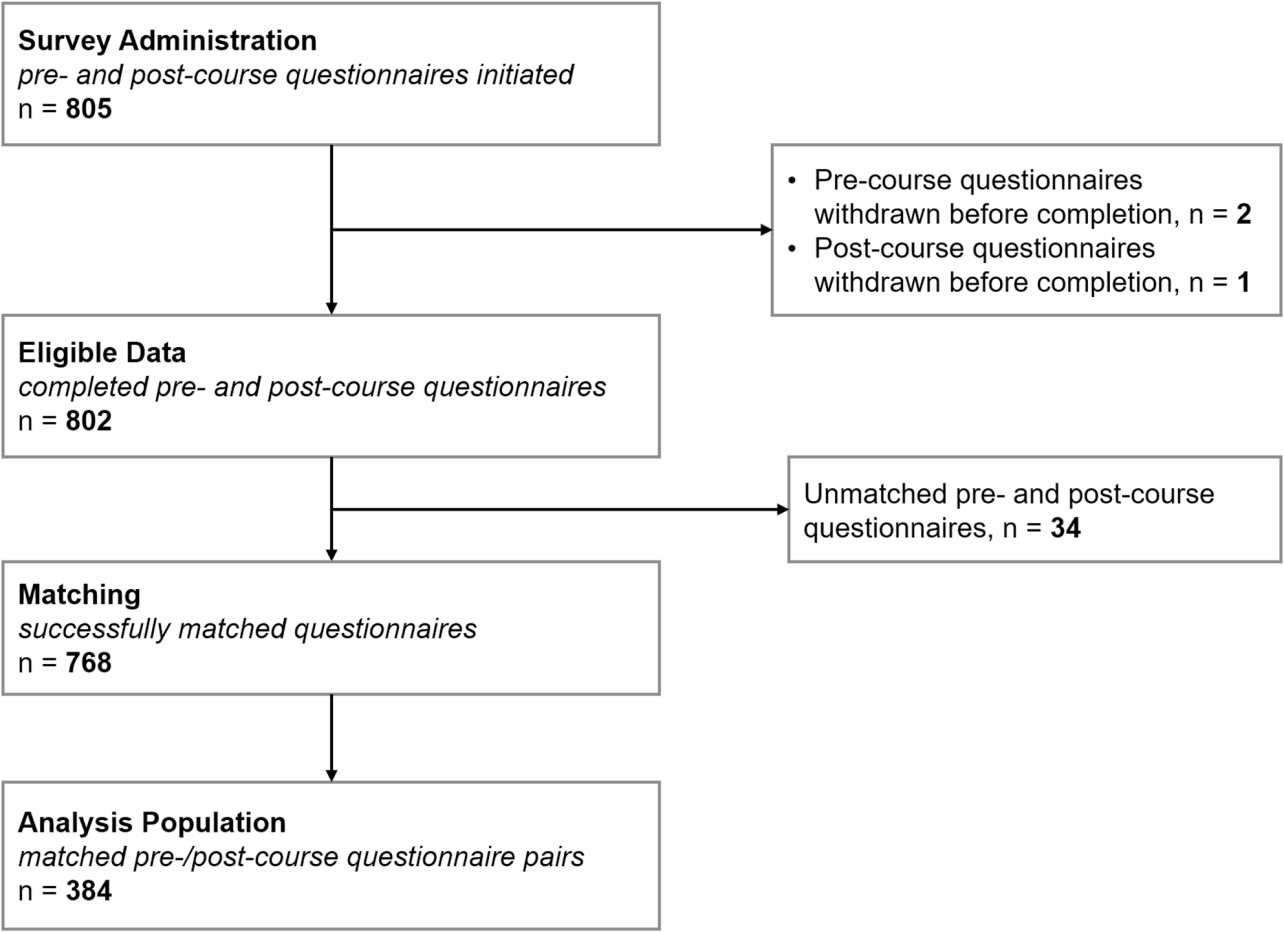
Study flow diagram in accordance with STROBE [18]

A total of 412 students participated in the emergency medicine course and were invited to complete study questionnaires; of these, 97.3% (401) completed both the pre- and post-course surveys. After matching based on pseudonymized codes, 93.2% (384) were successfully assigned at the end of the study (Fig. 1).

### Characteristics of pre- and post-course questionnaire

64% of participants were female. The mean age of all participants was 23 (SD 2.4) years. In accordance with curricular allocation, the questionnaire data revealed that 90% were medical students and 10% were dental students.

25% (*n* = 95/384) of the participants had already completed prior medical training. 13% (*n* = 12/95) of these were active as first responders with the “Region of Lifesaver” initiative. Furthermore, 18% (68/384) of the students had previously performed CPR. Of these, 65% (*n* = 44/68) had medical training, whereas 35% (*n* = 24/68) had no prior medical training.

99.5% (*n* = 382/384) of the participants considered the course suitable for increasing confidence in performing BLS. Additionally, 95.3% (*n* = 366/384) regarded the course as appropriate preparation for participation in the “Region of Lifesavers” initiative (Table 1). Free-form text responses are presented in Table 2.

**Table 1:** Characteristics of pre- and post-course questionnaire (SD= standard deviation, BLS = basic life support, CPR = cardiopulmonary resuscitation)

Pre-Course Questionnaire		
Questions	Response options	Results
Gender	Male	35.9% (138/384)
	Female	64.1% (246/384)
	Diverse (non-binary)	0% (0/384)
Age	In years (SD)	23 (2.4)
Degree course	Human medicine (medical students)	90.4% (347/384)
	Dentistry (dental students)	9.6% (37/384)
Have you already completed medical training?	Yes	24.7% (95/384)
	No	75.3% (289/384)
If yes (Single-Choice):	EMS (all qualifications)	41.1% (39/95)
	Nursing (all qualifications)	24.2% (23/95)
	Physiotherapy	5.2% (5/95)
	Midwife	1.1% (1/95)
	Other	28.4% (27/95)
Do you already prefer a specialization?	Yes	43.8% (168/384)
	No	56.2% (216/384)
If yes (Single-Choice):	Surgery	22.6% (38/168)
	Internal medicine	11.3% (19/168)
	Acute medicine (anesthesia/critical care)	12.5% (21/168)
	Gynecology	11.3% (19/168)
	Pediatrics	11.3% (19/168)
	Dentistry	10.7% (18/168)
	Research	1.2% (2/168)
	Other	19.1% (32/168)
Choose your training level for resuscitation: (Single-Choice)	No course to date	1.0% (4/384)
	Only one course to date (e.g. first aid)	55.5% (213/384)
	Already attended several courses	33.9% (130/384)
	I am a BLS trainer	1.0% (4/384)
	I am a professional	8.6% (33/384)
Choose reasons for insufficient training: (Single-Choice)	No courses available	15.6% (60/384)
	Not relevant for me	5.0% (19/384)
	No time capacities Courses are too expensive	44.5% (171/384) 12.8% (49/384)
	Other reasons	4.4% (17/384)
	No reason (adequate training level)	17.7% (68/384)
Would you like to have more training?	Yes	95.8% (368/384)
	No	4.2% (16/384)
Have you already provided resuscitation on a real patient?	Yes	17.7% (68/384)
	No	82.3% (316/384)
How do you rate your skills in resuscitation?	Scale 1–10 (1 = worst, 10 = best) mean, (SD)	4.9 (1.8)
Are you already registered with “Region of Lifesavers”?	No	95.1% (365/384)
	Yes	4.9% (19/384)
	Yes, installed and active	3.1% (12/384)
	Yes, but uninstalled/deleted the app	1.8 (7/384)
The app is active on my smartphone (multiple choice)	Not yet called out to an operation	17% (2/12)
	Received at least one alert	67% (8/12)
	At least one operation involving patient contact	17% (2/12)
	At least one operation and performed CPR	17% (2/12)
	Eligible for the next operation	42% (5/12)
I do not use the app/ no longer use the app (multiple choice)	Due to technical difficulties	14% (1/7)
	App alerts are too disruptive	43% (3/7)
	Wish to leave the project	0% (0/7)
	Due to negative experiences in an operation	0% (0/7)
	Other reasons	43% (3/7)
Are you satisfied with the course? (yes/no)	Yes	99.5% (382/384)
	No	0.5% (2/384)
How do you rate your skills in resuscitation?	Scale 1–10 (1 = worst, 10 = best) mean, (SD)	7.6 (1.2)
Do/Did you feel comfortable with the idea of helping a person suffering from cardiac arrest?		
Before the Emergency Medicine course	Scale 1–10 (1 = worst, 10 = best) mean, SD	3.9 (2.3)
After the Emergency Medicine course	Scale 1–10 (1 = worst, 10 = best) mean, SD	7.9 (1.5)
The following aspect gives me the greatest confidence to undertake action: (Single-Choice)	A better level of knowledge	15.1% (58/384)
	Sense of situational control	6.8% (26/384)
	Better understanding of pathophysiology	0.8% (3/384)
	Practical training	77.3% (297/384)
The following aspect is most likely to contribute to uncertainty to undertake action: (Single-Choice)	Fear of doing something wrong	31.5% (121/384)
	Stress	13.6% (52/384)
	Feeling of not being able to	15.6% (60/384)
	Lack of practice/training	39.3% (151/384)
Do you intend to participate as a first responder? (Single-Choice)	Yes	76.0% (292/384)
	No	19.8% (76/384)
	I am already registered	4.2% (16/384)
If yes: Which aspect is most likely to contribute to your decision? (Single-Choice)	Willingness to help	48.3% (141/292)
	The topic is important	22.6% (66/292)
	Feeling of making a difference	9.6% (28/292)
	I feel able to do this	18.5% (54/292)
	Other factors play a role	1.0% (3/292)
If no: Which aspect is most likely to contribute to your decision? (Single-Choice)	I don’t want to participate	6.7% (5/75)
	Too stressful	37.3% (28/75)
	I don’t feel it is useful	0% (0/75)
	I don’t feel I can do it	41.3% (31/75)
	Other factors play a role	14.7% (11/75)
The course is suitable … to feel more confident in the potential use of BLS than before	Yes	99.5% (382/384)
	No	0.5% (2/384)
The course is suitable … to prepare students for participation in “Region of Lifesavers”	Yes	95.3% (366/384)
	No	4.7% (18/384)

**Table 2:** Free-form text response options are presented in this table. Results from optional comments are provided in the Supplement

Question (pre-course questionnaire)
**“**Choose reasons for insufficient training**” **(*n* = 17)
➢ Too lazy so far
➢ Too little integrated into other activities, e.g., school lessons
➢ Regular practice would be important
➢ I hope we’ll learn that later
➢ Lack of time in everyday work
➢ Haven’t engaged with it
➢ Not offered by the employer
➢ Forgotten
➢ Not on the radar
➢Quickly forgotten
➢ No longer employed
➢ Convenience
➢ Lacking consistent practice
➢ Regrettably haven’t made an effort
➢ Own fault and too little promotion
➢ Studies
➢ No easily accessible option

Question (pre-course questionnaire)
“I do not use the app/no longer use the app… other reasons” (*n* = 2)
➢ Fear of insufficient competence
➢ Because I do not feel qualified enough

Question (post-course questionnaire)
“Do you intend to participate as a first responder? If yes: Which aspect is most likely to contribute to your decision? Other factors play a role” (*n* = 3)
➢ It is important and life-saving
➢ Due to the lack of medical resources in rural areas
➢ To bridge the time until emergency services arrive in order to improve the outcome

Question (post-course questionnaire)
**“**Do you intend to participate as a first responder? If no: Which aspect is most likely to contribute to your decision? Other factors play a role” (*n* = 6)
➢ I would like more practice before registering. I do not feel sufficiently prepared yet
➢ Time
➢ I’m still unsure and need to think about it a bit more
➢ I don’t have time
➢ Little time
➢ My phone battery is too weak

### Pre- vs. post-course comparison

**Fig. 2:**
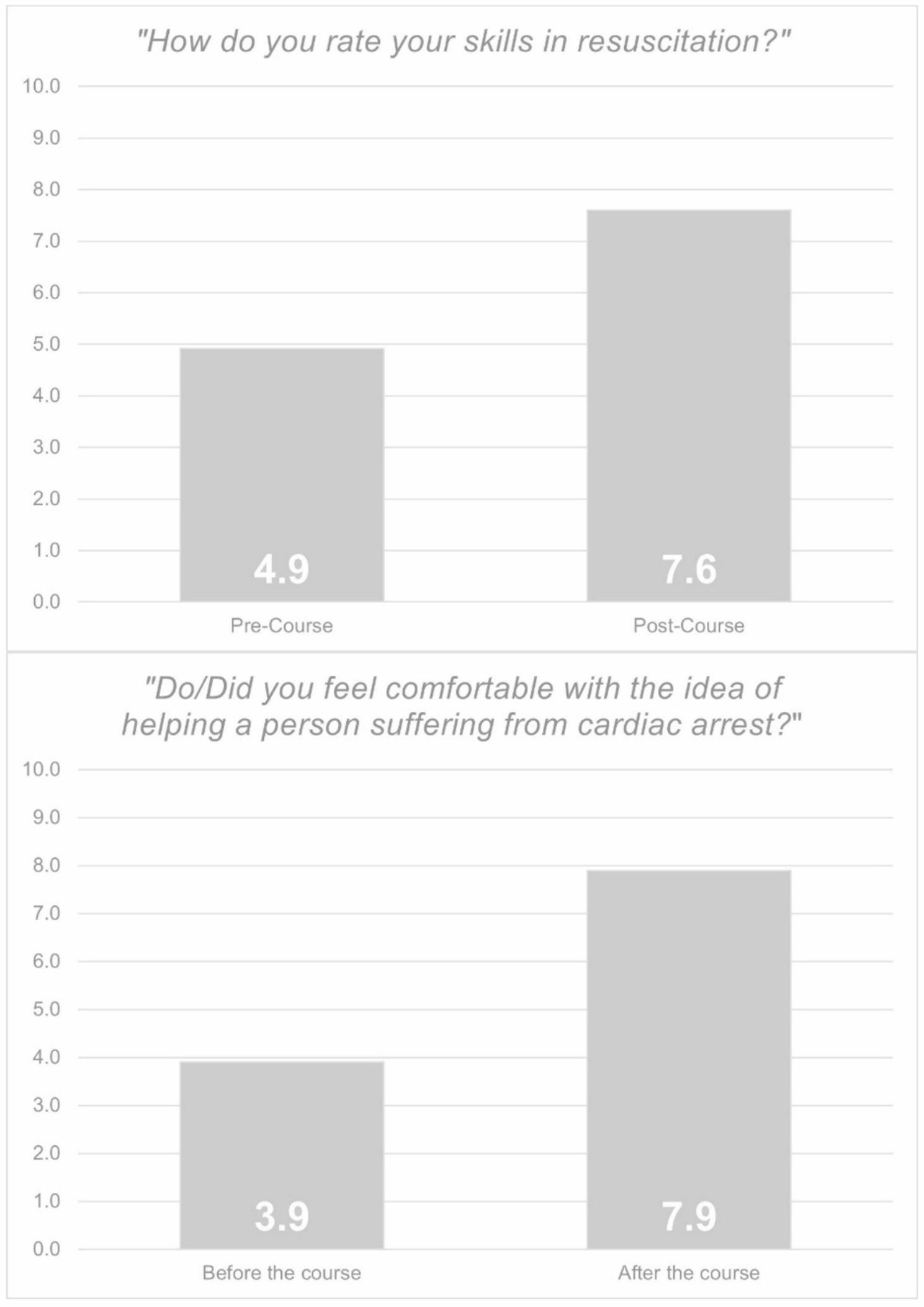
Pre- vs. Post-Course comparison. Above: The question “*How do you rate your skills in resuscitation?*” was included in both the pre-course and post-course questionnaires and was based on a Likert scale ranging from 0 (worst) to 10 (best) (*p* < 0.001). Below: The question “*Do/Did you feel comfortable with the idea of helping a person suffering from cardiac arrest?*” was assessed using a Likert scale from 0 (worst) to 10 (best) in the post-course questionnaire (*p* < 0.001)

The question “*How do you rate your skills in resuscitation?*” was included in both the pre-course and post-course questionnaires and was based on a Likert scale ranging from 0 (worst) to 10 (best). Participants rated their skills prior to the course at 4.9 (SD 1.8) and after the course at 7.6 (SD 1.2) (*p* < 0.001) (Fig. 2).

The question “*Do/Did you feel comfortable with the idea of helping a person suffering from cardiac arrest?*” was assessed using a Likert scale from 0 (worst) to 10 (best) in the post-course questionnaire, with participants reflecting on their self-perceived comfort before and after the emergency medicine course. Prior to the course, participants reported a mean score of 3.9 (SD 2.3), whereas following completion of the course, the mean increased to 7.9 (SD 1.5) (*p* < 0.001) (Fig. 2).

### Participation as first responder

The dichotomized endpoint, *“Intention to participate as a first responder”*, was indicated positively by 80.2% of participants (n = 308) and negatively by 19.8% (n = 76). The primary aspect underlying the decision to participate was *“Willingness to help”* (48.3%), followed by *“*The topic is important” (22.6%). Conversely, the primary aspects underlying the decision against participation were “I don’t feel I can do it” (41.3%) and “Too stressful” (37.3%).

In a binary logistic regression analysis with a dichotomized endpoint and dichotomized predictors, medical students enrolled in Human Medicine, a pre-course self-rated resuscitation skill level > 5, and a post-course comfort level > 5 regarding helping a person with cardiac arrest were identified as predictors of the intention to participate as a first responder. The model was statistically significant (χ²(3) = 27.12, *p* < 0.001) and model fit was adequate (Hosmer–Lemeshow *p* = 0.889). Detailed results are presented in Table 3.

**Table 3:** Participation as a first responder: binary logistic regression was performed to assess factors associated with the dichotomized outcome “Intention to participate as a first responder.” The table presents regression coefficients (B), standard errors (SE), *p*-values, odds ratios (Exp(B)), and 95% confidence intervals (CI). The model was statistically significant (χ² (3) = 27.12, *p* < 0.001) and model fit was adequate (Hosmer–Lemeshow *p* = 0.889)

Item	Regression coefficient	Standard error	*p*-value	Exp(B)	95% confidence interval for Exp(B)
Degree course: Human medicine	0.90	0.39	0.02	2.47	1.15–5.30
How do you rate your skills in resuscitation? > 5 (pre-course)	0.95	0.32	0.03	2.59	1.39–4.82
Do/Did you feel comfortable with the idea of helping a person suffering from cardiac arrest? > 5 (after the course)	1.42	0.44	0.01	4.13	1.73–9.83
Constant	-0.98	0.57	0.09	0.38	-

## Discussion

The present study demonstrates a notably high level of self-reported motivation among medical students to engage as first responders. An overwhelming majority regarded the course format as well-suited for preparing participants to take part in the “Region of Lifesavers” initiative. The principal aim of this work was to investigate which motivational factors encourage medical students to register and participate as community first responders, and to identify the barriers that may prevent such engagement.

Almost half of the respondents reported “willingness to help” as their primary motivation for becoming a first responder, thereby corroborating findings from previous findings [15]. This was followed by “the topic is important,” which was cited by almost a quarter of participants. The importance of minimizing delays to the first chest compression through the implementation of smartphone alerting systems was evidently conveyed during the course, and this modern concept appears to have played a convincing role in motivating students to consider first responder involvement. The aspect of “participation as a first responder” was indicated by over 80% of participants, reflecting a high level of willingness and corroborating findings by Taracamaz et al. who reported similarly elevated engagement rates of over 90% in their student cohort [15].

The primary reason cited against participation was *“I don’t feel I can do it”*, reported by 41% of respondents, closely followed by *“Too stressful”*. At this point, it is important to emphasize that the proportion of participants who categorically could not imagine participating as first responders comprised only about 20%. Thus, while perceived inability and stress represent prominent barriers, they affect only a minority of respondents. Nevertheless, despite their medical education and prior exposure to emergency medicine, residual psychological barriers persist, indicating that even medically trained individuals are not immune to hesitation in real-world emergency situations. Addressing the factors *“I don’t feel I can do it”* and *“Too stressful”* in a meaningful way remains challenging. Students’ perceptions of inadequate ability (“I don’t feel I can do it”) reflect low self-efficacy, which is associated with avoidance of demanding responsibilities [19]. Additionally, anticipating the role as excessively stressful (“Too stressful”) aligns with cognitive stress appraisal models, whereby tasks perceived as exceeding available coping resources are less likely to be undertaken [20]. In contrast to the general public, reluctance among medical students appears to be less driven by a lack of theoretical knowledge and more by concerns related to performance expectations, role perception, and perceived preparedness. Together, low self-efficacy and high anticipated stress may reduce students’ willingness to register as first responders. However, potential strategies to mitigate this uncertainty are supported by the study’s own findings. When asked *“The following aspect gives me the greatest confidence to undertake action”*, an overwhelming majority of participants identified *“Practical training”* (77%). This finding is further reinforced by an inverse response pattern to the question *“The following aspect is most likely to contribute to uncertainty to undertake action”*, which was predominantly answered with *“Lack of practice/training”*. These results clearly show that practical training is the main modifiable factor influencing confidence and perceived stress. When considering why the desired level of training is not achieved, the most frequently cited barrier to sufficient training was *“No time capacities”*. Notably, the desire for more training was expressed almost unanimously across the entire cohort, with 96% of all students indicating a strong wish for additional training opportunities. This underscores a clear mismatch between high motivation and limited structural feasibility and time resources.

A particularly promising finding in the context of high motivation to participate as first responders is that only 5% of participants were registered prior to attending the emergency medicine course. This suggests that there is substantial untapped potential with regard to both the target group and the educational setting. Strictly speaking, formal registration as a first responder is only possible after completion of an emergency medicine course. However, when focusing specifically on students with prior medical training, the proportion already engaged as first responders remained low, with only 13% (12/95) reporting active involvement. The reasons for this are likely multifactorial and may include limited awareness of first responder systems, insufficient promotion, or the absence of a concrete and facilitated registration process [14]. With respect to participants who were previously or currently registered as first responders but discontinued their engagement *“Due to technical difficulties”* or due to *“App alerts are too disruptive”*, it should be noted that this subgroup was very small (19 registered individuals of 384 participants in the study), limiting the interpretability of these findings. These findings suggest that structural and system-level factors, rather than individual motivation, play a decisive role and need to be addressed through improvements in system design and implementation [6].

Finally, when examining the performance and training effectiveness of the course structure, self-assessed resuscitation skills showed a marked increase, rising from a mean score of 4.9 to 7.6 on a 10-point Likert scale. While this result must be interpreted with caution -given that perceived self-efficacy tends to increase following successful training- it is consistent with findings from previous simulation-based studies [21–23]. A relevant question concerns the duration of this effect, which would also be pertinent for registration outcomes. Following the establishment of the proposed concept, actual registration behavior among medical students after completion of the course should therefore be examined. A similar pattern was observed for the item *“Do/Did you feel comfortable with the idea of helping a person suffering from cardiac arrest?”*, which increased substantially from 3.9 to 7.9, further supporting the positive impact of structured practical training. In this context, curriculum planners are considering complementing the existing third-year training with a further course or examination in the fifth year, which would support sustained competence and confidence within a longitudinal training concept.

In summary, the present findings highlight that medical students exhibit high motivation to act as first responders, and that structured, practical training effectively enhances both self-efficacy and confidence in resuscitation tasks. While individual barriers such as perceived inability and stress remain relevant, they affect a minority and can be mitigated through targeted educational strategies.

### Limitations

Participants were asked to self-assess their confidence levels, which may introduce a self-reporting bias or discalibration due to under- and overconfidence effects. Medical students may have overestimated or underestimated their own abilities, leading to potential discrepancies between perceived and actual competence. Such subjective evaluations, while valuable for understanding self-perception, do not necessarily reflect objective skill level or motivation (objective skill evaluation during the course itself was performed using CPR feedback systems as part of the practical training). They also may not be sustainable with need for further evaluation of the effect that might change over time without stimulating exposition to emergencies or emergency training. Therefore, the results should be interpreted with caution. Additionally, social desirability may have played a role in this survey. Furthermore, the study intentionally included all students, including those who were already registered as community first responders. This decision was made in acknowledgment of the fact that future cohorts are also likely to contain students with prior registration. As such, the focus was not only on the motivation to register, but also on the motivation to remain actively engaged in the program. Therefore, a strict distinction between newly recruited participants and those who were already registered was not enforced. This should be taken into account when interpreting the findings.

## Conclusion

Effective management of cardiac arrest requires innovative strategies such as structured first responder systems. In order to maximize available personal resources, it is crucial to engage with all individuals with medical training. This study underscores a strong motivation among medical students for first responder engagement and highlights the crucial role of practical training in fostering confidence and willingness to act. The emergency medicine course holds significant potential — not only by targeting the appropriate audience but also by providing an optimal platform for preparing medical students to serve as first responders. These findings underscore the importance of incorporating such training initiatives into medical curricula to fully harness the potential of medical students and better meet the needs of modern cardiac arrest response systems.

## Supplementary Information

Supplementary Material 1.

## Data Availability

Reasonable requests to provide raw data material will be assessed by contacting the corresponding author.

## Declarations

### Language check

During the preparation of this work the authors used Microsoft Word Editor and ChatGPT (OpenAI, GPT-5.2) to check language and readability. After using this service, the authors reviewed and edited the content as needed and take full responsibility for the content of the publication.

## Abbreviations

AED: Automated External Defibrillator
BLS: Basic Life Support
CPR: Cardio Pulmonary Resuscitation
EMS: Emergency Medical Service
OHCA: Out-of-Hospital Cardiac Arrest
SAS: Smartphone Alerting System
SD: Standard deviation
