# Supplementary material for: Motivational aspects among MEDical Students to Participate as first ResPONDer in out-of-hospital cardiac arrest (MEDSPOND): a pre-post educational intervention study
Source: BMC Med Educ. 2026 May 14;26:766. doi: 10.1186/s12909-026-09445-8 (PMC13179622; doi:10.1186/s12909-026-09445-8)
Supplement: Supplementary file 1 — Supplementary Material 1. [file 12909_2026_9445_MOESM1_ESM.docx]

**Supplement**

**Pre-course questionnaire**

| **1** | Gender | M/W/D |
| --- | --- | --- |
| **2** | Year of birth | YYYY |
| **3** | Degree course | Human medicine  Dentistry |
| **4** | Have you already completed medical training? | Yes  No |
| **4a** | If yes (Single-Choice): | EMS (all qualifications)  Nursing (all qualifications)  Physiotherapy  Midwife  Other |
| **5** | Do you already prefer a specialization? | Yes  No |
| **5a** | If yes (Single-Choice): | Cardiology  Surgery  Gynecology  Internal medicine  Acute medicine (anesthesia-emergency medicine-intensive care)  Pediatrics  Dentistry  Research  Other |
| **6** | Choose your training level for resuscitation: (Single-Choice) | No course to date  Only one course to date (e.g. first aid)  Already attended several courses  I am a BLS trainer  I am a professional |
| **7** | Choose reasons for insufficient training: (Single-Choice) | No courses  Not relevant for me  No time capacities  Courses are too expensive  Other reasons  No reason (adequate training level) |
| **8** | Would you like to have more training? | Yes  No |
| **9** | Have you already provided resuscitation on a real patient? | Yes  No |
| **10** | How do you rate your skills in resuscitation? | Scale 1-10 |
| **11** | Are you already registered with “Region of Lifesavers”? | No  Yes, the app is installed and active on my smartphone.  Yes, but I have uninstalled/deleted the app. |
| **12** | The app is active on my smartphone and I...  (multiple choice) | ...has not yet been called out to an incident  ...have received at least one alert so far  ...has had at least one assignment involving patient contact to date  ...has had at least one call-out to date and performed resuscitation measures  ...would also go on the next mission |
| **13** | I do not use the app/no longer use the app...  (multiple choice) | ...because there are technical difficulties with it  ...because the app is too disruptive (e.g. loud alerts in inappropriate situations)  ...because I no longer wish to be part of the project.  ...because I have had negative experiences in an operation.  ...for other reasons (free-form text) |

**Post-course questionnaire**

| **1** | Are you satisfied with the course? (yes/no) | Yes  No |
| --- | --- | --- |
| **2** | How do you rate your skills in resuscitation? | Scale 1-10 |
| **3** | Do/Did you feel comfortable with the idea of helping a person suffering from cardiac arrest? |  |
| **3a** | Before the Emergency Medicine course | Scale 1-10 |
| **3b** | After the Emergency Medicine course | Scale 1-10 |
| **4** | The following aspect gives me the greatest **confidence** to undertake action: (Single-Choice) | A better level of knowledge  Sense of situational control  Better understanding of pathophysiology  Practical training |
| **5** | The following aspect is most likely to contribute to **uncertainty** to undertake action: (Single-Choice) | Fear of doing something wrong  Stress  Feeling of not being able to  Lack of practice/training |
| **6** | Do you intend to participate as a first responder? (Single-Choice) | Yes  No  I am already registered |
| **7** | If **yes**: Which aspect is most likely to contribute to your decision? (Single-Choice) | Willingness to help  The topic is important  Feeling of making a difference  I feel able to do this  Other factors play a role (free-form text) |
| **8** | If **no**: Which aspect is most likely to contribute to your decision? (Single-Choice) | I don't want to participate  Too stressful  I don't feel it is useful  I don't feel I can do it  Other factors play a role (free-form text) |
| **9** | The course is suitable ...  to feel more confident in the potential use of BLS than before | Yes  No |
| **10** | The course is suitable ...  to prepare students for participation in “Region of Lifesavers” | Yes  No |
| **11** | Are there any other aspects you would like to share with us? (optional) | Free-form text |

**- Original German Version -**

**Pre-course Fragebogen**

| **1** | Geschlecht | M/W/D |
| --- | --- | --- |
| **2** | Geburtsjahr | YYYY |
| **3** | Studiengang | Humanmedizin  Zahnmedizin |
| **4** | Haben Sie bereits eine medizinische Ausbildung abgeschlossen? | Ja  Nein |
| **4a** | Falls ja (Single-Choice): | Rettungsdienst (alle Qualifikationen)  Pflege (alle Qualifikationen)  Physiotherapie  Geburtshelfer/in  Andere |
| **5** | Präferieren Sie bereits eine Fachrichtung? | Ja  Nein |
| **5a** | Falls ja (Single-Choice): | Chirurgie  Innere Medizin  Akutmedizin (Anästhesie-Intensivmedizin)  Gynäkologie  Pädiatrie  Zahnmedizin  Forschung  Andere |
| **6** | Wählen Sie Ihr Trainingslevel für die Wiederbelebung: (Single-Choice) | Bisher kein Kurs  Bisher nur ein Kurs (z.B. Erste-Hilfe)  Schon mehrere Kurse  Ich bin BLS Trainer  Ich bin professionelle/r Helfer/in |
| **7** | Wählen Sie Gründe für nicht ausreichendes Training: (Single-Choice) | kein Angebot  für mich keine Relevanz  keine zeitlichen Kapazitäten  Kurse sind zu teuer  andere Gründe  kein Grund (adäquates Trainingsniveau) |
| **8** | Würden Sie sich mehr Training wünschen? | Ja  Nein |
| **9** | Haben Sie Wiederbelebungsmaßnahmen bereits beim realen Patienten durchgeführt? | Ja  Nein |
| **10** | Wie schätzen Sie Ihre Kompetenzen bei der Wiederbelebung ein? | Scala 1-10 |
| **11** | Sind Sie bereits bei "Region der Lebensretter" registriert? | Nein  Ja, die App ist auf meinem Smartphone installiert und aktiv  Ja, aber ich habe ich habe die App deinstalliert/gelöscht |
| **12** | Die App ist auf meinem Smartphone aktiv und ich...  (Mehrfachauswahl) | …wurde bisher nicht zu einem Einsatz alarmiert  …habe bisher mindestens eine Alarmierung erhalten  …hatte bisher mindestens einen Einsatz mit Patientenkontakt  …hatte bisher mindestens einen Einsatz und führte Wiederbelebungsmaßnahmen durch  …würde auch zum nächsten Einsatz gehen |
| **13** | Ich nutze die App nicht oder nicht mehr...  (Mehrfachauswahl) | …weil es technische Schwierigkeiten damit gibt  …weil die App zu "aufdringlich" ist (z.B. laute Alarmierung in ungeeigneten Situationen)  …weil ich nicht mehr Teil des Projekts sein möchte  …weil ich schlechte Erfahrungen im Einsatz gemacht habe  …aus anderen Gründen (offene Eingabe) |

**Post-course Fragebogen**

| **1** | Sind Sie mit dem Kurs zufrieden? (ja/nein) | Ja  Nein |
| --- | --- | --- |
| **2** | Wie schätzen Sie Ihre Kompetenzen bei der Wiederbelebung ein? | Scala 1-10 |
| **3** | Fühlen/Fühlten Sie sich wohl bei der Vorstellung, einer Person mit Herz-Kreislaufstillstand zu helfen? |  |
| **3a** | Vor dem Kurs QB Notfallmedizin | Scala 1-10 |
| **3b** | Nach dem Kurs QB Notfallmedizin | Scala 1-10 |
| **4** | Folgender Aspekt gibt mir am ehesten **Sicherheit**, die Maßnahmen durchzuführen: (Single-Choice) | Besserer Wissensstand  Gefühl, die Situation zu beherrschen  Besseres Verständnis der Pathophysiologie  Praktisches Training |
| **5** | Folgender Aspekt trägt am ehesten zur **Unsicherheit** bei, um die Maßnahmen durchzuführen: (Single-Choice) | Angst etwas falsch zu machen  Stress  Gefühl, nicht in der Lage zu sein  Fehlende Praxis/Training |
| **6** | Beabsichtigen Sie eine Teilnahme als Ersthelfer/in? (Single-Choice) | Ja  Nein  Ich bin bereits registriert |
| **7** | Falls **ja**: Welcher Aspekt trägt am ehesten zu Ihrer Entscheidung bei? (Single-Choice) | Bereitschaft zu helfen  Das Thema ist wichtig  Gefühl, einen Unterschied zu machen  Ich fühle mich in der Lage dazu  Andere Faktoren spielen eine Rolle (+Freitext) |
| **8** | Wenn **nein**: Welcher Aspekt trägt am ehesten zu Ihrer Entscheidung bei? (Single-Choice) | Ich möchte an diesem Projekt nicht teilnehmen  Zu stressig  Das Gefühl, dass es nicht nützlich ist  Ich fühle mich nicht in der Lage  Andere Faktoren spielen eine Rolle (Freitext) |
| **9** | Der Kurs ist geeignet, …  um sich in der potentiellen Anwendung von BLS sicherer zu fühlen, als zuvor | Ja  Nein |
| **10** | Der Kurs ist geeignet, …  um Studierende auf eine Mitwirkung bei „Region der Lebensretter“ vorzubereiten | Ja  Nein |
| **11** | Gibt es weitere Aspekte, die Sie uns mitteilen möchten? (optional) | Freitext |

**Optional comment results**

| **Question (post-course questionnaire)**  **“**Are there any other aspects you would like to share with us? (optional)**” (n= 93)** |
| --- |
| - Super organization. It was a lot of fun. Thank you!! - Great organization, especially the transition between practice and theory. - I thought the course was amazing! Great learning atmosphere, good instruction, and it was very good to have the alternation between theory and practice. Having theory in the morning and practice in the afternoon would have been more tiring. - Very interesting - I thought it was great - I found the course module really well structured and very well organized! Great teaching in the seminars and practical sessions! Thank you so much! - Excellent, keep it up! - It was great! - Great course with valuable content - Friendly, helpful tutors/instructors - A very good course! The best so far! - I really liked having the chance to practice these situations, instead of just learning the theory. - Great course!!! - Very well-structured course with lots of practice and good theory sections, thank you! - Well organized, very nice instructors - The alternation between theory and practice is good - Very great presenters—competent, open, and very friendly! Keep it up - Very helpful in a short time! Thank you - Really very well designed and instructive course, nice instructors, pleasant atmosphere! - Friendly and competent instructors - Very good sequence/organization - Tip-top, but a longer lunch break would be nice, ours was only 30 minutes - Recommend the video "Freiburger Rettungskette" - Bad break times, rather one long one - Great course, very practical and educational training! Good mix of theory and practice - Motivating, instructive, friendly team, realistic training. Best course in my studies so far - Small groups were ideal, instructors were very good - More feedback during the practical exercises would have been helpful for learning - No - Nice course, no joke - Everything perfect - Maybe consider splitting the course over 3 days to keep motivation for practical training high - Great course, practice is so important—practicing again and again improves performance for every first responder or professional - Very well designed and memorable - If possible, more training opportunities should be available - No - Very good course - It was fun, especially the case scenarios in the practical blocks - Top - Ideally, I’d like to spend a few more hours on the ambulance - By far the best course in the 5th semester. Had a lot of fun. - One of the best courses in the studies so far! Huge compliment! - Really cool course, thanks - It was a lot of fun :) - Very good instructors - More frequent repetition and refreshers would be great! Two days over the whole degree are too few - Lots of practice was great - Very pleasant learning atmosphere, very nice instructors - Great course, I enjoyed it - Exam as a team -> Show communication and teamwork skills - More training, more skill stations - It was a great course. The alternation between theory and case scenarios kept one’s attention steady, and the material stuck better in general, thank you!! - No - One of the best courses so far in the program. Please repeat such practical training in later semesters as well - Really good :) Maybe make the resuscitation session longer for more team rotations so everyone gets more routine - I would like to have refreshers in the future, because I don’t feel well enough prepared yet. If we had practical sessions once a month (also under stress), I would install the app, but at the moment I don’t know how I’d react under stress. - Would like to practice even more realistic scenarios—once in the stairwell or similar - In my opinion, the course could be offered over a longer period, e.g., a week - Great teaching! - Thank you for the great course and the commitment from the teaching team! Sometimes things were a bit fast for me, so another day would be good - Very successful course, especially the constant room changes made it easier to stay focused and kept attention levels high ;) - No - The alternation of practice and theory is very good - Switching between practice and theory prevented overload by information. I liked that we were allowed to make mistakes and learn from them, because elsewhere in the degree program this is not common. - It was good that the exercises were repeated and we got to rotate roles. This way, everyone got involved and it really helped internalize the learning! - Great variety between practice and theory, so you take a lot away - The combination of alternating practice and theory is great. You can apply knowledge directly and clarify questions from practice. Best holiday course so far! - Would be happy to practice even more. - Very well managed with lots of practice! Many thanks!! - Thank you for the practice, great course!! - This course came at a very unsuitable time. The basic resuscitation—yes, that’s worth it and should be done regularly. But anything beyond layperson resuscitation I consider unnecessary at this point. - Too little practical training, since it was only 2 days - More feedback, especially suggestions for improvement - Would prefer having it earlier in the program - Very exciting and varied course! - No negative criticism: No added value for those with prior training; practice too unstructured, too few mandatory formats - I am now registered for "Region der Lebensretter" - Thank you for this great course, I really liked the alternation between seminars and case scenarios and I think, given the available time, I learned a lot. I also liked that we got the lectures and slides in advance to prepare at home. - Technical issues with the dummies/The Lifepack didn’t work. Too few skill stations before the case scenarios. Without prior experience in EMS, you get thrown in at the deep end with a team that doesn’t even know the equipment. - I thought the course was very good - Cool course - Great seminar - I think it was great that we had so many opportunities to practice ;) - Sometimes outstanding teaching from a didactic point of view - The many repetitions were great, and there was always feedback during practical training, which I never had before - Practicing reading heart activity, but the course day shouldn't be during the semester break - It was well structured, which I really appreciated - I found the practical exercises very meaningful, they give you confidence, thank you! - Very pleasant alternation between theory and practice - It was fun. Well structured. Very helpful. Thank you! - Great practical part. Thank you. Lectures could focus more on the ABCDE scheme - I found the practical and theory sessions very helpful and exciting. You could learn a lot. Thanks again for the course. - Didactically excellent with alternating theory and practical sections |

**List of Items used for binary log. regression**

| Item1 | Male | No/Yes |
| --- | --- | --- |
| Item2 | Female | No/Yes |
| Item3 | Degree course: Human medicine | No/Yes |
| Item4 | Degree course: Dentistry | No/Yes |
| Item5 | Have you already completed medical training? | No/Yes |
| Item6 | EMS (all qualifications) | No/Yes |
| Item7 | Nursing (all qualifications) | No/Yes |
| Item8 | Physiotherapy | No/Yes |
| Item9 | Midwife | No/Yes |
| Item10 | Other medical training | No/Yes |
| Item11 | Training level: No course to date | No/Yes |
| Item12 | Training level: Only one course to date (e.g. first aid) | No/Yes |
| Item13 | Training level: Already attended several courses | No/Yes |
| Item14 | Training level: I am a BLS trainer | No/Yes |
| Item15 | Training level: I am a professional | No/Yes |
| Item16 | Have you already provided resuscitation on a real patient? | No/Yes |
| Item17 | How do you rate your skills in resuscitation? > 5 (pre-course) | No/Yes |
| Item18 | How do you rate your skills in resuscitation? > 5 (post-course) | No/Yes |
| Item19 | Do/Did you feel comfortable with the idea of helping a person suffering from cardiac arrest? (**before** the course) > 5 | No/Yes |
| Item20 | Do/Did you feel comfortable with the idea of helping a person suffering from cardiac arrest? (**after** the course) > 5 | No/Yes |
